# Association of child labor with risk and protective factors for Chronic Noncommunicable Diseases in Brazilian schoolchildren: National School Health Survey 2015

**DOI:** 10.1590/1980-549720230012.supl.1

**Published:** 2023-04-21

**Authors:** Alan Cristian Marinho Ferreira, Alanna Gomes da Silva, Crizian Saar Gomes, Deborah Carvalho Malta

**Affiliations:** I Universidade Federal de Minas Gerais, Nursing School, Postgraduate Program in Nursing – Belo Horizonte (MG), Brazil.; II Universidade Federal de Minas Gerais, Nursing School, Department of Maternal Child and Public Health Nursing – Belo Horizonte (MG), Brazil.; III Universidade Federal de Minas Gerais, Medical School, Postgraduate Program in Public Health– Belo Horizonte (MG), Brazil.

**Keywords:** Child labor, Teenagers, Chronic non-communicable diseases, Risk factors, Protective factors

## Abstract

**Objective:**

To analyze the sociodemographic profile of adolescents working in Brazil and the association of child labor with risk and protection factors for Chronic Noncommunicable Diseases.

**Methods:**

Cross-sectional study with data from sample 2 of the 2015 National School Health Survey (PeNSE). The variables gender, age, ethnicity/skin color, administrative dependence on school and maternal education, eating habits, physical activity and drug use were analyzed by prevalence and respective 95% confidence intervals (95%CI) and calculation of crude and adjusted Odds Ratio.

**Results:**

A total of 10,926 students participated in the survey, of which 16.9% (95%CI 15.1–18.9) were currently working/employed. Child labor was higher among male adolescents (ORa: 1.82; 95%CI 1.55–2.15); aged between 16 and 17 years (ORa: 2.96; 95%CI 2.37–3.69); enrolled in public schools (ORa: 1.69; 95%CI 1.14–2.52); whose mothers had incomplete high school (ORa: 1.54; 95%CI 1.11–2.13); living in the South region of the country (ORa: 2.17; 95%CI 1.60–2.94). Adolescents who worked were more likely to smoke (ORa: 1.94; 95%CI 1.52–2.48); use alcohol (ORa: 2.01; 95%CI 1.71–2.36) and drugs (ORa: 1.76; 95%CI 1.35–2.31); perform physical activity (ORa: 1.24; 95%CI 1.07–1.44); consume sweets (ORa: 1.30; 95%CI 1.13–1.49), fried snacks (ORa: 1.41; 95%CI 1.15–1.74), and soft drinks (ORa: 1.23; 95%CI 1.06–1.44); however, they were less likely to present sedentary behavior (ORa: 0.68; 95%CI 0.59–0.79).

**Conclusion:**

Child labor in Brazil is related to sociodemographic differences. Those who worked were more likely to show risk behaviors for NCDs, but they were more physically active.

## INTRODUCTION

Child labor is characterized by the early entry of an individual into the labor market, that is, before the age of 18; for the most part, it occurs informally and illegally, exposing them to precarious conditions and no rights offered by labor laws^1,2^.

In several countries, including Brazil, child labor is prohibited, except for the condition of minor apprentice, allowed from the age of 14^3^. However, even with the prohibition, in 2015 about 168 million children and adolescents aged between 5 and 17 years were active in the labor market^4^ globally, while in Brazil, it accounted for 1.8 million children/adolescents^5^.

Child labor can be the result of different settings of vulnerability and socioeconomic inequalities in which children and adolescents are inserted^2^ and perpetuate these cycles of vulnerabilities, since it causes school dropout, making it impossible for them to take higher education courses and get better jobs and wages in adult life^6,7^.

When entering the labor market, children and adolescents can also be exposed to occupational risks inherent to activities^8^, which directly impact physical and mental health, and contribute to the adoption of unhealthy lifestyles, making them more susceptible to developing non-communicable chronic diseases (NCDs) throughout life^7^.

A study carried out with data from the National School Health Survey (PeNSE) in 2012 showed that working Brazilian adolescents were more likely to smoke, use alcohol and illicit drugs, and get involved in situations of violence^9^. However, the monitoring of working conditions in childhood and adolescence and their influence on risk and protection factors for CNCDs, such as eating habits, physical activity and use of licit and illicit drugs, must be continuous to enable the implementation of more effective health policies^7,10^. In addition, it is important to investigate the sociodemographic conditions of these workers so that these policies are specific and capable of meeting the needs of this population. Thus, a more current overview of the profile of Brazilian adolescents who work is expected to be shown and contribute to the creation of public health policies, social protection, inspection and restraint of this practice, when illegal, with view to promoting health and preventing diseases.

This study aimed to analyze the sociodemographic profile of adolescents working in Brazil and the association of child labor with risk and protective factors for CNCDs.

## METHODS

### Study Design

This was a cross-sectional study that used data from the PeNSE survey, carried out in 2015.

### Scenary

PeNSE is the broadest survey carried out in Brazil aimed at investigating factors related to the health of adolescents and mapping of behaviors and life habits^11^.

The research sample was gathered by conglomerate in two stages, where the schools are configured as the first stage and the group classes are the second, with all students chosen to answer the questionnaire present on the day of the research^11^. Data was collected from students throughout the national territory^11^. More information about PeNSE is available and can be consulted in other publications^11,12^.

### Data Collection and Time Frame

The PeNSE survey was conducted between April and September 2015. Data was collected using a self-administered questionnaire made available on a mobile device (smartphone used specifically to carry out surveys by the Brazilian Institute of Geography and Statistics)^11^.

### Participants

Sample 2 of the survey was used in this study, which analyzed data from 371 schools, 653 classes and 16,608 questionnaires; however, since data was collected with all students in person, questionnaires filled in by students outside the age range of 13 to 17 years were excluded^11^. Thus, the final sample included data from 10,926 students between the ages of 13 and 17, regularly enrolled and attending the 6th-9th grades of elementary school (formerly 5th to 8th grades), and 1st-3rd grades of high school in public and private institutions in Brazil^11^.

### Study Variables

The present study used the variable “child labor”, through the question: “Do you currently have a job, work or business?”, whose answer options were “yes” and “no”. Adolescents who answered “yes” were framed within child labor.

The sociodemographic variables used in this study were: biological sex (male and female); age range (from 13 to 15 years old, from 16 to 17 years old); skin color (white, brown, black, others); school network (public, private); maternal educational level (incomplete primary education, incomplete secondary education, incomplete higher education, completed higher education, does not know or not informed); and region (North, Northeast, Southeast, South, Midwest).

Variables related to risk and protective factors for NCDs were:

1. Eating habits: regular consumption of healthy and unhealthy food markers:
  - Regular consumption of fresh fruit: “In the past seven days, on how many days did you eat fresh fruit?”
  - Regular consumption of greens and/or vegetables: “In the last seven days, on how many days did you eat at least one type of vegetable? Examples: lettuce, pumpkin, broccoli, onion, carrot, chayote, cabbage, spinach, cucumber, tomato, etc. Do not include potatoes and manioc (cassava).”
  - Regular consumption of fried snacks: “In the last seven days, on how many days did you eat fried snacks? Example: french fries (not counting potato chips) or fried snacks such as “coxinha”, fried kibbeh, fried pastry, acarajé, etc.”
  - Regular consumption of ultra-processed/industrialized foods: “In the last seven days, on how many days did you eat industrialized/ultra-processed salty foods, such as hamburgers, ham, mortadella, salami, sausages, instant noodles, packaged snacks, salty crackers?”
  - Regular consumption of soft drinks: “In the last seven days, how many days did you drink soda?”
  - Regular consumption of sweets: “In the last seven days, on how many days did you eat sweets (desserts, candies, chocolate, gum, bonbons or lollipops)?”
    All questions had as answer options: zero days in the last seven days, one day in the last seven days, two days in the last seven days, three days in the last seven days, four days in the last seven days, five days in the last seven days, six days in the last seven days, and every day in the last seven days. Regular consumption was considered when the adolescent reported consumption on five or more days a week^11^.
2. Physical activity: practice of weekly physical activity for enough time and sedentary behavior:
  - Physically active: “In the last seven days, on how many days did you do physical activity for at least 60 minutes (one hour) a day? (Add up all the time you spent doing any type of physical activity each day): zero days in the last seven days (zero days), one day in the last seven days, two days in the last seven days, three days in the last seven days, four days in the last seven days, five days in the last seven days, five days plus Saturday in the last seven days, five days plus Saturday and Sunday in the last seven days”.
    Those who reported at least 300 minutes of any type of physical activity during the last seven days prior to the survey were considered physically active^11^.
3. Sedentary behavior: “On a typical weekday, how long do you spend sitting down, watching television, using the computer, playing video games, talking with friends or doing other activities sitting down? (Not counting Saturday, Sunday, holidays and sitting time at school): Up to one hour a day, more than one up to two hours a day, more than two up to three hours a day, more than three up to four hours a day, more than four to five hours a day, more than five to six hours a day, more than six to seven hours a day, more than seven to eight hours a day, more than eight hours a day”.
  Sedentary behavior was defined as spending at least three hours a day sitting down on an average week.
4. Use of legal and illegal drugs: variables that investigate regular use, defined as the use of these substances on at least one day in the last 30 days prior to the survey^11^:
  - Currently smoke: “In the last 30 days, on how many days did you smoke cigarettes?”
  - Currently alcohol use: “In the last 30 days, on how many days did you drink at least one glass or one dose of alcoholic beverage (a dose is equivalent to a can of beer, or a glass of wine, or a dose of cachaça or whiskey, etc.)?”
  - Current illicit drug use: “In the last 30 days, how many days did you use drugs such as marijuana, cocaine, crack, cola, “loló”, “lança-perfume”, ecstasy, oxy, etc.?”

All questions related to licit and illicit drugs had as answer options: never in the last 30 days (zero days), one or two days in the last 30 days, three to five days in the last 30 days, six to nine days in the last 30 days, 10 to 19 days in the last 30 days, 20 to 29 days in the last 30 days, every day in the last 30 days.

### Data analysis

Descriptive analysis of the variables was carried out using prevalence and respective 95% confidence intervals (95%CI), and logistic regression was used to calculate the crude and adjusted odds ratios (OR) by sex, continuous age, school network and region.

In the sociodemographic analysis, the variable “child labor” was used as a response variable. In the analysis of association between child labor and risk and protection factors for CNCDs, the variable “child labor” was considered an explanatory variable. Results whose p-value was less than or equal to 0.05 were considered significant.

Analyses were performed using the Data Analysis and Statistical Software Stata pakage, version 16.0, using the module “survey”, which considers post-stratification weights.

### Ethical Aspects

PeNSE 2015 was approved by the National Ethics Research Committe of the Ministry of Health, Opinion No. 1,006,467. Students received guidance on the research and were able to decide about their participation by answering the question “Dear student, do you agree to participate in this research? Yes, No”^11^.

## RESULTS

In total, 10,926 adolescent schoolchildren participated in PeNSE, of which 16.9% (95%CI 15.1–18.9) performed some type of work. Male adolescents (ORa: 1.82; 95%CI 1.55–2.15), aged between 16 and 17 years (ORa: 2.96; 95%CI 2.37–3.69), who attended public schools (ORa: 1.69; 95%CI 1.14–2.52), whose mothers have low educational level (incomplete primary education (ORa: 1.45; 95%CI 1.05–2.01); incomplete high school (ORa: 1.54; 95%CI 1.11–2.13) and living in the South (ORa: 2.17; 95%CI 1.60–2.94) and Midwest regions (ORa: 1.65; 95%CI 1.18–2.30) were more likely to work (Table 1).

**Table 1:** Prevalence of child labor among Brazilian adolescents according to sociodemographic variables and *odds ratio*. National School Health Survey, 2015.

		Workers (%) (95%CI)	Crude OR (95%CI)	Adjusted OR* (95%CI)
Total		16.7 (15.1–18.9)	-	-
Male		20.7 (18.4–23.2)	1.73 (1.47–2.04)	1.82 (1.55–2.15)
Female		13.1 (11.3–15.2)	Ref	Ref
Age group (years)				
	13–15	11.1 (10.0–12.4)	Ref	Ref
	16–17	26.3 (22.5–30.5)	2.85 (2.28–3.55)	2.96 (2.37–3.69)
Ethnicity/Skin color				
	White	18.4 (16.0–21.0)	Ref	Ref
	Brown	15.1 (13.4–17.00	0.79 (0.68–0.92)	0.92 (0.79–1.06)
	Black	19.0 (15.4–23.2)	1.04 (0.81–1.34)	1.10 (0.86–1.41)
	Others	16.8 (12.8–21.7)	0.90 (0.65–1.24)	1.08 (0.77–1.51)
School network				
	Public	17.8 (15.8–19.9)	1.72 (1.09–2.71)	1.69 (1.14–2.52)
	Private	11.2 (7.6–16.1)	Ref	Ref
Mother's educational level				
	Incomplete primary education	19.3 (16.4–22.7)	1.60 (1.19–2.16)	1.45 (1.05–2.01)
	Incomplete high school	20.4 (17.2–23.9)	1.71 (1.27–2.31)	1.54 (1.11–2.13)
	Incomplete higher education	17.7 (15.1–20.6)	1.43 (1.10–1.87)	1.26 (0.95–1.68)
	Complete higher education	13.0 (10.2–16.5)	Ref	Ref
	Does not know/not informed	13.9 (12.2–15.8)	1.08 (0.80–1.46)	1.17 (0.85–1.63)
Region				
	North	14.5 (11.6–18.0)	Ref	Ref
	Northeast	12.7 (10.2–15.7)	0.85 (0.60–1.21)	0.83 (0.57–1.21)
	Southeast	16.6 (13.3–20.6)	1.17 (0.82–1.68)	1.18 (0.83–1.68)
	South	26.0 (22.2–30.3)	2.07 (1.52–2.83)	2.17 (1.60–2.94)
	Midwest	21.0 (17.4–25.3)	1.57 (1.13–2.18)	1.65 (1.18–2.30)

* Adjusted OR: odds ratio adjusted for sex, age, administrative department of the school and region. Ref: reference group for the calculation crude and adjusted OR.

As for the association between child labor and risk and protective factors for CNCDs, schoolchildren who worked were more likely to smoke (ORa: 1.94; 95%CI 1.52–2.48); consume alcoholic beverages (ORadj: 2.01; 95%CI 1.71–2.36); use illicit drugs (ORa: 1.76; 95%CI 1.35–2.31); being physically active (ORadj: 1.24; 95%CI 1.07–1.44); consume sweets (ORa: 1.30; 95%CI 1.13–1.49); consume fried snacks (ORa: 1.41; 95%CI 1.15–1.74); and consume soft drinks (ORa: 1.23; 95%CI 1.06–1.44). On the other hand, working adolescents were less likely to have a sedentary behavior (ORa: 0.68; 95%CI 0.59–0.79) (Table 2).

**Table 2:** Association between child labor and risk and protective factors for chronic noncommunicable diseases in Brazilian adolescents. National School Health Survey, 2015.

	Workers			
	% No^a^ (95%CI)	% Yes^b^ (95%CI)	Crude OR (95%CI)a/b	Adjusted OR* (95%CI)
Cigarette use in the last 30 days	5.47 (4.8–6.22)	11.8 (9.9–14.1)	2.32 (1.84–2.92)	1.94 (1.52–2.48)
Alcohol intake in the last 30 days	25.9 (24.2–27.8)	45.9 (42.5–49.4)	2.43 (2.08–2.83)	2.01 (1.71–2.36)
Use of illicit drugs in the last 30 days	4.52 (3.9–5.24)	9.8 (8.0–11.9)	2.28 (1.77–2.94)	1.76 (1.35–2.31)
Physically active	18.8 (17.7–20.0)	24.1 (21.8–26.5)	1.37 (1.18–1.59)	1.24 (1.07–1.44)
Sedentary behavior	54.3 (52.2–56.4)	46.4 (43.2–49.5)	0.73 (0.63–0.84)	0.68 (0.59–0.79)
Regular consumption of fruits and vegetables	18.3 (16.9–19.8)	20.1 (17.2–23.3)	1.12 (0.91–1.38)	1.15 (0.93–1.43)
Regular consumption of sweets	39.8 (38.1–41.5)	44.4 (41.3–47.5)	1.21 (1.06–1.38)	1.30 (1.13–1.49)
Regular consumption of fried snacks	13.2 (12.1–14.4)	17.1 (14.9–19.7)	1.36 (1.12–1.64)	1.41 (1.15–1.74)
Regular consumption of soft drinks	26.4 (24.6–28.3)	31.5 (28.8–34.5)	1.29 (1.11–1.48)	1.23 (1.06–1.44)
Regular consumption of ultra-processed/industrialized foods	31.4 (29.5–33.5)	32.9 (29.9–33.5)	1.07 (0.90–1.26)	1.06 (0.90–1.25)

a: denominator used to calculate the odds ratio.;

b: numerator used to calculate the odds ratio;

a/b: formula used to calculate the odds ratio;

* Adjusted OR: odds ratio adjusted for sex, age, school network and region.

## DISCUSSION

This study showed that 16.9% of the adolescents carried out some type of work, the majority of them being males aged between 16 and 17, enrolled in public schools, whose mother had the lowest levels of education, and residents of the South and Midwest regions of the country. Adolescents who worked were more likely to smoke, consume alcoholic beverages, use illicit drugs, perform physical activity and consume unhealthy foods such as sweets, fried snacks and soft drinks. On the other hand, they were less likely to have sedentary behavior.

Early exposure to work can be determining in maintaining socioeconomic vulnerability, as it reduces the opportunity to attend school and remain in it, consequently contributing to lower wages in adult life, in addition to reducing the ability to positively respond to problems and cope with adversities in adult life^13,14^. Furthermore, child labor places adolescents in an environment full of adults, exposing them to habits that are incompatible with their age group and which contributes to a phenomenon known as “child adultization”, which can lead to early maturation and adoption of unhealthy habits^15,16^.

Regarding the differences between sexes, most societies around the world adopt a division of labor determined by gender, in which women tend to carry out housework and men assume the role of guaranteeing family subsistence^17^. The higher prevalence of child labor among males may be related to the reproduction of this tradition by families, especially the most vulnerable ones, in which boys are assigned to work outside the home and girls assume the responsibilities and tasks at home^18^. It should also be noted that, in addition to working at home, girls are still subject to work in other homes, often without any type of remuneration^17,18^.

From the age of 15, an individual becomes part of the economically active population and, in the case of low-income families, the need for better living conditions increases^19^. This explains the fact that most of the working students assessed in this study were older, between 16 and 17 years old, suggesting that they could be in search of financial autonomy in the face of growing consumer demand.

When considering school networks and maternal education as a proxy for socioeconomic status, the majority of those who workare part of the most vulnerable population. Public schools have a history of meeting social demands of the population, dealing with issues such as vulnerability^20^, and, as child labor is associated with contexts like this, it is understandable that most woeking children were enrolled in public schools, as they are more demanded by low-income families^2^. Regarding maternal education, some studies have reported an association between the mothers‘ education and child labor, demonstrating that higher levels of maternal education are a protective factor against child labor, as there is the understading of the importance of education for a better quality of life^21,22^ and better opportunities in the future, valuing education to the detriment of work^18^.

With regard to regional differences, child labor is known to be strongly influenced by cultural aspects, and in many of these cultures, offering child labor is considered a form of education^22^. The higher prevalence of child labor in the southern region of the country may be linked to these cultural factors, but also to differences in population density between the biggest regions of the country and to the low dynamism of productive activities in the north and northeast regions compared to Center-South regions of the country^19,22^. It is also worth mentioning that the findings of this study on the predominance of child labor by region conflict with other information available in the literature, which show that child labor ism ore common in rural regions, thus requiring a deeper understanding of this theme to better grasp these differences^19,22^.

Regarding higher chances of using licit drugs (cigarettes and alcoholic beverages) and illicit drugs (marijuana, cocaine, crack, cola, “loló”, “lança-perfume”, ecstasy or other) among adolescents who worked evidenced in this study, it is believed to be associated with the effect of childhood adultization, since they start to have contact with older people and, because they want to be part of the group, they end up adopting their behaviors, including drug use and active sex life^16^.

As for tobacco, it is known to be one of the main risk factors for the development of CNCDs; even so, in Brazil, in 2019, 22.6% (95%CI 21.7–23.4) of adolescents had already tried cigarettes at least once in their lives^23,24^. Smoking in adolescence is associated with worse economic conditions, such as receiving one to three minimum wages and being enrolled in public network educational institutions^23,25^, in addition to increasing the chance of using other substances^23^.

With regard to alcoholic beverages, it is important to highlight that, among adolescents, it is also related to a culture of socialization, leisure activities, pursuit of pleasure, reduction of shyness and acceptance of friends^26^, and that this practice has immediate and future negative repercussions for health such as effects on memory, risk of accidents and external injuries, unprotected sexual intercourse, influence on mood and CNCDs in adult life^27^.

Regarding the use of illicit drugs, the literature points to the rupture of social and family ties, depression and conditions of vulnerability such as income inequalities and poverty among the causes of consumption of these substances^28^, in addition to the effect of early life maturation that working adolescents go through^29,30^. The consequences can lead to cognitive, emotional and social impairments^31^.

Regarding practice of physical activity for enough time reported by adolescents who worked, it is believed that this scenario can be explained by the type of work carried out by these individuals, who sometimes work in places such as farms, industries and nursing homes, which require greater movement and use of force^32^. It is noteworthy that the physical activity indicator used in this study encompasses all domains of physical activity, as recommended by the World Health Organization for research among schoolchildren. The reduction in sedentary behavior, that is, sitting for three or more hours a day, may be associated with the decrease in time available to carry out activities that contribute to a sedentary lifestyle, such as using computers, cell phones and watching television^33^.

Working schoolchildren were also more likely to consume unhealthy foods such as sweets, fried snacks and soft drinks. This is similar what adult workers experience, with prioritization of foods that are more practical to prepare, have lower prices, and low nutritional value^34,35^. Some authors have already highlighted the importance of implementing public policies aimed at healthy eating, valuing the traditional Brazilian food culture, and of proposing regulatory measures that help build a healthy eating environment^36^.

The main limitation of this study is that PeNSE investigates only students regularly enrolled and attending public and private schools in Brazil, leaving out those who do not have this educational bond^13,37^, which, in general, are still more vulnerable and may be framed in child labor as well. Despite this, PeNSE remains representative of the school population in the country, being an important component for the health surveillance system for Brazilian adolescents^37^.

In 2015, 16.9% of the students performed some type of work, and this practice was more commonly reported by older boys enrolled in public school, whose mothers had lower educational levels, and living in the South and Midwest regions of the country. These individuals were more likely to present risk factors for CNCDs, such as drug use and consumption of unhealthy foods, but the scenarios of physical activity and reduction of sedentary time were more a favorable.

The findings of this study show the importance of monitoring the behavior of Brazilian students, since exposure to risk factors in childhood can increase the chances of developing some type of CNCD in adult life. Evidence points to immediate and future risks that child labor can bring to the health of adolescents and reinforces the need for immediate action by the State to fight this and reduce social vulnerabilities and poverty.
