# Supplementary material for: Association of child labor with risk and protective factors for Chronic Noncommunicable Diseases in Brazilian schoolchildren: National School Health Survey 2015
Source: Rev Bras Epidemiol. 2023 Apr 21;26(Suppl 1):e230012. doi: 10.1590/1980-549720230012.supl.1 (PMC10176732; doi:10.1590/1980-549720230012.supl.1)
Supplement: Translation

# Translation: Associação do trabalho infantil com fatores de risco e proteção para doenças crônicas não transmissíveis em escolares brasileiros: Pesquisa Nacional de Saúde do Escolar 2015

**DOI:** 10.1590/1980-549720230012.supl.1.1

**Authors:** Alan Cristian Marinho Ferreira, Alanna Gomes da Silva, Crizian Saar Gomes, Deborah Carvalho Malta

**Affiliations:** I Universidade Federal de Minas Gerais, Escola de Enfermagem, Programa de Pós-Graduação em Enfermagem – Belo Horizonte (MG), Brasil.; 11 Universidade Federal de Minas Gerais, Escola de Enfermagem, Departamento de Enfermagem Materno Infantil e Saúde Pública – Belo Horizonte (MG), Brasil.; III Universidade Federal de Minas Gerais, Faculdade de Medicina, Programa de Pós-Graduação em Saúde Pública – Belo Horizonte (MG), Brasil.

**Keywords:** Trabalho infantil, Adolescentes, Doenças crônicas não transmissíveis, Fatores de risco, Fatores de proteção

## Abstract

**Objetivo:**

Analisar o perfil sociodemográfico dos adolescentes que trabalham no Brasil e a associação do trabalho infantil com fatores de risco e proteção para doenças crônicas não transmissíveis.

**Métodos:**

Estudo transversal com dados da amostra 2 da Pesquisa Nacional de Saúde do Escolar de 2015. Analisaram-se as variáveis sexo, idade, raça/cor da pele, dependência administrativa da escola e escolaridade materna, variáveis acerca de alimentação, atividade física e uso de drogas. Realizaram-se análises por meio das prevalências e respectivos intervalos de confiança de 95% (IC95%) e cálculo da *odds ratio* bruta e ajustada.

**Resultados:**

Participaram da pesquisa 10.926 escolares, destes, 16,9% (IC95% 15,1–18,9) trabalhavam. O trabalho infantil foi maior nos adolescentes: do sexo masculino (ORaj: 1,82; IC95% 1,55–2,15); idade entre 16 e 17 anos (ORaj: 2,96; IC95% 2,37–3,69); que estudavam em escolas públicas (ORaj: 1,69; IC95% 1,14–2,52); com escolaridade materna igual ao ensino médio incompleto (ORaj: 1,54; IC95% 1,11–2,13); residentes da região Sul (ORaj: 2,17; IC95% 1,60–2,94). Esses adolescentes trabalhadores apresentaram maiores chances de: fumar (ORaj: 1,94; IC95% 1,52–2,48); consumir bebidas alcoólicas (ORaj: 2,01; IC95% 1,71–2,36); usar drogas ilícitas (ORaj: 1,76; IC95% 1,35–2,31); realizar atividade física (ORaj: 1,24; IC95% 1,07–1,44); consumir guloseimas (ORaj: 1,30; IC95% 1,13–1,49); consumir salgados fritos (ORaj: 1,41; IC95% 1,15–1,74), e refrigerantes (ORaj: 1,23; IC95% 1,06–1,44). Contudo apresentaram menor chance de comportamento sedentário (ORaj: 0,68; IC95% 0,59–0,79).

**Conclusão:**

Houve diferenças sociodemográficas em relação ao trabalho infantil no Brasil. Os que trabalhavam apresentaram maiores chances de manifestar comportamentos de risco para as DCNT, no entanto eram mais ativos fisicamente.

## INTRODUÇÃO

O trabalho infantil é caracterizado pela entrada precoce do indivíduo no mercado de trabalho, ou seja, antes dos 18 anos, e, em sua maioria, ocorre informal e ilegalmente, expondo-o a condições precárias e sem os direitos oferecidos pelas leis trabalhistas^1,2^.

Em diversos países, incluindo o Brasil, o trabalho infantil é proibido, com exceção da condição de menor aprendiz, que se inicia a partir dos 14 anos^3^. Entretanto, mesmo com a proibição, globalmente, em 2015, cerca de 168 milhões de crianças e adolescentes entre 5 e 17 anos atuavam no mercado de trabalho^4^ e, no Brasil, em 2019, esse número foi de 1,8 milhão^5^.

O trabalho infantil pode ser resultado dos diversos contextos de vulnerabilidade e desigualdades socioeconômicas em que as crianças e os adolescentes estão inseridos^2^ e consegue perpetuar esses ciclos de vulnerabilidades, uma vez que provoca a evasão escolar, impossibilitando-os de realizar cursos superiores e obter melhores empregos e salários na vida adulta^6,7^.

Ao adentrarem no mercado de trabalho, as crianças e os adolescentes também podem se expor aos riscos ocupacionais inerentes às atividades realizadas^8^, os quais impactam diretamente na saúde física e mental, além de contribuir para a adoção de estilos de vida não saudáveis, tornando-os mais suscetíveis ao desenvolvimento de doenças crônicas não transmissíveis (DCNT) ao longo da vida^7^.

Um estudo realizado com os dados da Pesquisa Nacional de Saúde do Escolar (PeNSE) em 2012 evidenciou que os adolescentes brasileiros que trabalhavam apresentaram maiores chances de fumar, de consumir bebidas alcoólicas, de usar drogas ilícitas e de se envolver em situações de violência^9^. Contudo o monitoramento das condições de trabalho na infância e na adolescência e da sua influência sobre os fatores de risco e de proteção para as DCNT, como alimentação, prática de atividade física e uso de drogas lícitas e ilícitas, devem ser contínuos para possibilitar a implantação de políticas de saúde mais eficazes^7,10^. Além disso, torna-se importante investigar as condições sociodemográficas desses trabalhadores a fim de que essas políticas sejam específicas e capazes de atender às necessidades dessa população. Assim, espera-se fornecer um panorama mais atual do perfil dos adolescentes brasileiros que trabalham e contribuir com a criação de políticas públicas de saúde, de proteção social, de fiscalização e coibição dessa prática, quando ilegal, com vistas à promoção da saúde e à prevenção de agravos.

Este estudo teve como objetivos: analisar o perfil sociodemográfico dos adolescentes que trabalham no Brasil e a associação do trabalho infantil com fatores de risco e proteção para as DCNT.

## MÉTODOS

### Delineamento do estudo

Este foi um estudo transversal com utilização dos dados da PeNSE, realizada no ano de 2015.

### Cenário

A PeNSE é o mais amplo inquérito realizado no Brasil voltado para a investigação de fatores relacionados à saúde dos adolescentes do país e o mapeamento de comportamentos e hábitos de vida^11^.

A amostra da pesquisa é por conglomerado em dois estágios, as escolas se configuram como o primeiro estágio e as turmas dessas escolas o segundo, estando todos os alunos presentes no dia da realização da pesquisa eleitos para responderem ao questionário^11^. A pesquisa coletou dados de estudantes em todo o território nacional^11^. Mais informações acerca da PeNSE já estão disponíveis e podem ser consultadas em outras publicações^11,12^.

### Coleta de dados e período

A PeNSE foi conduzida entre abril e setembro de 2015. A coleta de dados foi realizada por meio de questionário autoaplicável disponibilizado em um dispositivo móvel de coleta (*smartphone* utilizado especificamente para realização das pesquisas do Instituto Brasileiro de Geografia e Estatísticas)^11^.

### Participantes

Para este estudo, foi utilizada a amostra 2 da pesquisa que analisou os dados de 371 escolas, 653 turmas e 16.608 questionários respondidos, porém, devido ao fato de a coleta ter sido feita com todos os alunos presentes no dia, houve a exclusão dos questionários de alunos fora da faixa etária de 13 a 17 anos^11^. Assim, a amostra final contou com dados de 10.926 estudantes entre 13 e 17 anos regularmente matriculados e frequentes entre o 6° e o 9° ano do ensino fundamental (antigamente 5ᵃ a 8ᵃ série) e da 1ᵃ à 3ᵃ série do ensino médio, de escolas das redes de ensino pública e privada do Brasil^11^.

### Variáveis do estudo

O presente estudo utilizou a variável “trabalho infantil”, por meio da pergunta: “Você tem algum trabalho, emprego ou negócio atualmente?”, cujas opções de respostas eram “sim” e “não”. Considerou-se em trabalho infantil os adolescentes que responderam “sim”.

As variáveis sociodemográficas utilizadas neste estudo foram: sexo (masculino e feminino); faixa etária (de 13 a 15 anos, de 16 e 17 anos); cor da pele (branca, parda, preta, outros); dependência administrativa da escola (pública, privada); escolaridade materna (ensino fundamental incompleto, ensino médio incompleto, universitário incompleto, universitário completo, não sabe ou não quis informar); e região (Norte, Nordeste, Sudeste, Sul, Centro-Oeste).

As variáveis relacionadas aos fatores de risco e de proteção para as DCNT foram:

1. Alimentação: consumo regular de alimentos marcadores de alimentação saudável e não saudável:
  - Consumo regular de frutas frescas ou salada de frutas: “Nos últimos sete dias, em quantos dias você comeu frutas frescas ou salada de frutas?”
  - Consumo regular de verduras e(ou) legumes: “Nos últimos sete dias, em quantos dias você comeu pelo menos um tipo de legume ou verdura? Exemplos: alface, abóbora, brócolis, cebola, cenoura, chuchu, couve, espinafre, pepino, tomate, etc. Não inclua batata e aipim (mandioca/macaxeira).”
  - Consumo regular de salgados fritos: “Nos últimos sete dias, em quantos dias você comeu salgados fritos? Exemplo: batata frita (sem contar a batata de pacote) ou salgados fritos como coxinha de galinha, quibe frito, pastel frito, acarajé, etc.”
  - Consumo regular de ultraprocessados/industrializados: “Nos últimos sete dias, em quantos dias você comeu alimentos industrializados/ultraprocessados salgados, como hambúrguer, presunto, mortadela, salame, linguiça, salsicha, macarrão instantâneo, salgadinho de pacote, biscoitos salgados?”
  - Consumo regular de refrigerantes: “Nos últimos sete dias, em quantos dias você tomou refrigerante?”
  - Consumo regular de guloseimas: “Nos últimos sete dias, em quantos dias você comeu guloseimas (doces, balas, chocolates, chicletes, bombons ou pirulitos)?”
  Todas as perguntas anteriores tiveram como opções de resposta: zero dias nos últimos sete dias, um dia nos últimos sete dias, dois dias nos últimos sete dias, três dias nos últimos sete dias, quatro dias nos últimos sete dias, cinco dias nos últimos sete dias, seis dias nos últimos sete dias e todos os dias nos últimos sete dias. Considerou-se consumo regular quando o adolescente reportou o consumo em cinco ou mais dias na semana^11^.
2. Atividade física: prática de atividade física semanal por tempo suficiente e comportamento sedentário:
  - Ativos fisicamente: “Nos últimos sete dias, em quantos dias você fez atividade física por pelo menos 60 minutos (uma hora) por dia? (Some todo o tempo que você gastou em qualquer tipo de atividade física, em cada dia) Nenhum dia nos últimos sete dias (zero dia), um dia nos últimos sete dias, dois dias nos últimos sete dias, três dias nos últimos sete dias, quatro dias nos últimos sete dias, cinco dias nos últimos sete dias, cinco dias mais sábado, nos últimos sete dias, cinco dias mais sábado e domingo, nos últimos sete dias”.
    Consideraram-se ativos fisicamente aqueles que relataram pelo menos 300 minutos de prática de qualquer tipo de atividade física durante os últimos sete dias anteriores à pesquisa^11^.
3. Comportamento sedentário: “Em um dia de semana comum, quanto tempo você fica sentado(a), assistindo televisão, usando computador, jogando videogame, conversando com amigos(as) ou fazendo outras atividades sentado(a)? (Não contar sábado, domingo, feriados e o tempo sentado na escola) Até uma hora por dia, mais de uma hora até duas horas por dia, mais de duas horas até três horas por dia, mais de três horas até quatro horas por dia, mais de quatro horas até cinco horas por dia, mais de cinco horas até seis horas por dia, mais de seis horas até sete horas por dia, mais de sete horas até oito horas por dia, mais de oito horas por dia”.
  Definiu-se como comportamento sedentário os adolescentes que passaram pelo menos três horas por dia realizando atividades sentados durante uma semana comum.
4. Drogas lícitas e ilícitas: variáveis que investigam o uso regular, definido como o uso dessas substâncias em pelo menos um dia nos últimos 30 dias anteriores à pesquisa^11^:
  - Fumam atualmente: “Nos últimos 30 dias, em quantos dias você fumou cigarros?”
  - Consomem álcool atualmente: “Nos últimos 30 dias, em quantos dias você tomou pelo menos um copo ou uma dose de bebida alcoólica (uma dose equivale a uma lata de cerveja, ou uma taça de vinho, ou uma dose de cachaça ou uísque etc.)?”
  - Uso de drogas ilícitas atualmente: “Nos últimos 30 dias, quantos dias você usou droga como maconha, cocaína, *crack*, cola, loló, lança-perfume, *ecstasy, oxy* etc.?”
  Todas as perguntas anteriores, relacionadas a drogas lícitas e ilícitas, tiveram como opções de resposta: nenhum dia nos últimos 30 dias (zero dia), um ou dois dias nos últimos 30 dias, de três a cinco dias nos últimos 30 dias, de seis a nove dias nos últimos 30 dias, de 10 a 19 dias nos últimos 30 dias, de 20 a 29 dias nos últimos 30 dias, todos os dias nos últimos 30 dias.

### Análise dos dados

Realizou-se análise descritiva das variáveis por meio das prevalências e dos respectivos intervalos de confiança de 95% (IC95%) e utilizou-se a regressão logística para cálculo da *odds ratio* bruta (ORb) e ajustada (ORaj) por sexo, idade contínua, dependência administrativa da escola e região.

Na análise do perfil sociodemográfico, utilizou-se a variável “trabalho infantil” como variável resposta. Já na análise de associação do trabalho infantil com os fatores de risco e proteção para as DCNT, a variável “trabalho infantil” foi considerada variável explicativa. Consideraram-se significativos os resultados cujo valor-p fosse menor ou igual a 0,05.

As análises foram realizadas no *Data Analysis and Statistical Software* Stata, versão 16.0, empregando-se o módulo *survey*, que considera os pesos de pós-estratificação.

### Aspectos éticos

A PeNSE 2015 foi aprovada pela Comissão Nacional de Ética em Pesquisa para Seres Humanos do Ministério da Saúde, com o Parecer n° 1.006.467. Os alunos receberam orientações sobre a pesquisa e puderam decidir acerca de sua participação, respondendo à pergunta “Prezado(a) estudante, você concorda em participar dessa pesquisa? Sim, Não”^11^.

## RESULTADOS

Participaram da PeNSE 10.926 escolares adolescentes, destes, 16,9% (IC95% 15,1–18,9) realizavam algum tipo de trabalho. Os adolescentes do sexo masculino (ORaj: 1,82; IC95% 1,55–2,15), com idade entre 16 e 17 anos (ORaj: 2,96; IC95% 2,37–3,69), que frequentam escolas públicas (ORaj: 1,69; IC95% 1,14–2,52), aqueles cujas mães tem baixa escolaridade (ensino fundamental incompleto (ORaj: 1,45; IC95% 1,05–2,01); ensino médio incompleto (ORaj: 1,54; IC95% 1,11–2,13) e das regiões Sul (ORaj: 2,17; IC95% 1,60–2,94) e Centro-Oeste (ORaj: 1,65; IC95% 1,18–2,30) apresentaram maiores chances de trabalhar (Tabela 1).

**Tabela 1:** Prevalência de trabalho infantil em adolescentes brasileiros segundo variáveis sociodemográficas e *odds ratio*. Pesquisa Nacional de Saúde do Escolar, 2015.

		Trabalham (%) (IC95%)	ORbruta (IC95%)	ORaj* (IC95%)
Total		16,7 (15,1–18,9)	-	-
Masculino		20,7 (18,4–23,2)	1,73 (1,47–2,04)	1,82 (1,55–2,15)
Feminino		13,1 (11,3–15,2)	Ref	Ref
Faixa etária (anos)				
	13–15	11,1 (10,0–12,4)	Ref	Ref
	16–17	26,3 (22,5–30,5)	2,85 (2,28–3,55)	2,96 (2,37–3,69)
Raça/cor da pele				
	Branca	18,4 (16,0–21,0)	Ref	Ref
	Parda	15,1 (13,4–17,00	0,79 (0,68–0,92)	0,92 (0,79–1,06)
	Preta	19,0 (15,4–23,2)	1,04 (0,81–1,34)	1,10 (0,86–1,41)
	Outros	16,8 (12,8–21,7)	0,90 (0,65–1,24)	1,08 (0,77–1,51)
Dependência administrativa da escola				
	Pública	17,8 (15,8–19,9)	1,72 (1,09–2,71)	1,69 (1,14–2,52)
	Privada	11,2 (7,6–16,1)	Ref	Ref
Escolaridade materna				
	Ensino fundamental incompleto	19,3 (16,4–22,7)	1,60 (1,19–2,16)	1,45 (1,05–2,01)
	Ensino médio incompleto	20,4 (17,2–23,9)	1,71 (1,27–2,31)	1,54 (1,11–2,13)
	Universitário incompleto	17,7 (15,1–20,6)	1,43 (1,10–1,87)	1,26 (0,95–1,68)
	Universitário completo	13,0 (10,2–16,5)	Ref	Ref
	Não sabe ou não quis informar	13,9 (12,2–15,8)	1,08 (0,80–1,46)	1,17 (0,85–1,63)
Região				
	Norte	14,5 (11,6–18,0)	Ref	Ref
	Nordeste	12,7 (10,2–15,7)	0,85 (0,60–1,21)	0,83 (0,57–1,21)
	Sudeste	16,6 (13,3–20,6)	1,17 (0,82–1,68)	1,18 (0,83–1,68)
	Sul	26,0 (22,2–30,3)	2,07 (1,52–2,83)	2,17 (1,60–2,94)
	Centro-Oeste	21,0 (17,4–25,3)	1,57 (1,13–2,18)	1,65 (1,18–2,30)

* ORaj: *odds ratio* ajustada por sexo, idade, dependência administrativa da escola e região. Ref: grupo de referência para o cálculo da ORb e da ORaj.

Quanto à associação do trabalho infantil com os fatores de risco e proteção para as DCNT, observa-se que os escolares que trabalhavam apresentaram maiores chances de fumar (ORaj: 1,94; IC95% 1,52–2,48); de consumir bebidas alcoólicas (ORaj: 2,01; IC95% 1,71–2,36); de usar drogas ilícitas (ORaj: 1,76; IC95% 1,35–2,31); de serem ativos fisicamente (ORaj: 1,24; IC95% 1,07–1,44); de consumir guloseimas (ORaj: 1,30; IC95% 1,13–1,49); de consumir salgados fritos (ORaj: 1,41; IC95% 1,15–1,74); e de consumir refrigerantes (ORaj: 1,23; IC95% 1,06–1,44). Por outro lado, os adolescentes que trabalhavam apresentaram menor chance de ter comportamento sedentário (ORaj: 0,68; IC95% 0,59–0,79) (Tabela 2).

**Tabela 2:** Associação entre o trabalho infantil e fatores de risco e proteção para doenças crônicas não transmissíveis em adolescentes brasileiros. Pesquisa Nacional de Saúde do Escolar, 2015.

	Trabalho			
	% Não^a^ (IC95%)	% Sim^b^ (IC95%)	ORbruta (IC95%)a/b	ORaj* (IC95%)
Uso de cigarro nos últimos 30 dias	5,47 (4,8–6,22)	11,8 (9,9–14,1)	2,32 (1,84–2,92)	1,94 (1,52–2,48)
Consumo de bebida alcoólica nos últimos 30 dias	25,9 (24,2–27,8)	45,9 (42,5–49,4)	2,43 (2,08–2,83)	2,01 (1,71–2,36)
Uso de drogas ilícitas nos últimos 30 dias	4,52 (3,9–5,24)	9,8 (8,0–11,9)	2,28 (1,77–2,94)	1,76 (1,35–2,31)
Ativos fisicamente	18,8 (17,7–20,0)	24,1 (21,8–26,5)	1,37 (1,18–1,59)	1,24 (1,07–1,44)
Comportamento sedentário	54,3 (52,2–56,4)	46,4 (43,2–49,5)	0,73 (0,63–0,84)	0,68 (0,59–0,79)
Consumo regular de frutas e hortaliças	18,3 (16,9–19,8)	20,1 (17,2–23,3)	1,12 (0,91–1,38)	1,15 (0,93–1,43)
Consumo regular de guloseimas	39,8 (38,1–41,5)	44,4 (41,3–47,5)	1,21 (1,06–1,38)	1,30 (1,13–1,49)
Consumo regular de salgados fritos	13,2 (12,1–14,4)	17,1 (14,9–19,7)	1,36 (1,12–1,64)	1,41 (1,15–1,74)
Consumo regular de refrigerantes	26,4 (24,6–28,3)	31,5 (28,8–34,5)	1,29 (1,11–1,48)	1,23 (1,06–1,44)
Consumo regular de alimentos ultraprocessados/industrializados	31,4 (29,5–33,5)	32,9 (29,9–33,5)	1,07 (0,90–1,26)	1,06 (0,90–1,25)

a: denominador utilizado para o cálculo da *odds ratio*.;

b: numerador utilizado para o cálculo da *odds ratio*;

a/b: fórmula utilizada para cálculo da *odds ratio*;

* ORaj: *odds ratio* ajustada por sexo, idade, dependência administrativa da escola e região.

## DISCUSSÃO

O estudo mostrou que 16,9% dos adolescentes realizavam algum tipo de trabalho, sendo a maioria do sexo masculino, com 16 e 17 anos, estudantes de escolas públicas, cuja mãe possuía os menores níveis de escolaridade, e moradores da região Sul e Centro-Oeste do país. Os adolescentes que trabalhavam apresentaram maiores chances de fumar, consumir bebidas alcoólicas, usar drogas ilícitas, realizar atividade física e consumir alimentos não saudáveis, como guloseimas, salgados fritos e refrigerantes. Em contrapartida, tiveram menor chance de ter comportamento sedentário.

A exposição precoce ao trabalho pode ser uma condição determinante na manutenção da vulnerabilidade socioeconômica, uma vez que reduz a oportunidade de frequentar uma escola e de se manter nela, contribuindo consequentemente para o recebimento de menores salários na vida adulta, além de diminuir a capacidade de respostas positivas aos problemas e ao enfrentamento das adversidades na vida adulta^13,14^. Ademais, o trabalho infantil insere o adolescente em um ambiente de convívio com os adultos, expondo-os a hábitos incompatíveis com sua faixa etária, o que contribui para um fenômeno denominado “adultização infantil”, que pode levar a uma maturação precoce e à adoção de hábitos não saudáveis^15,16^.

Em relação às diferenças entre os sexos encontradas no presente estudo, grande parte das sociedades pelo mundo adotam uma divisão do trabalho determinada pelo gênero, em que as mulheres tendem a realizar os trabalhos domésticos e os homens assumem o papel de garantir a subsistência familiar^17^. A maior prevalência do trabalho infantil no sexo masculino pode estar relacionada com a reprodução desse costume pelas famílias, sobretudo aquelas mais vulneráveis, nas quais os meninos são designados ao trabalho fora de casa e as meninas assumem as responsabilidades do trabalho no lar^18^. Cabe ainda ressaltar que, além do trabalho no próprio lar, as meninas ainda estão sujeitas ao trabalho em outras casas, por vezes, sem nenhum tipo de remuneração^17,18^.

Quanto à idade, a partir dos 15 anos o indivíduo se torna parte da parcela populacional economicamente ativa e, no caso de famílias de baixa renda, aumenta a necessidade de procurar, no trabalho, uma forma de se obter melhores condições de vida^19^. Isso explica o fato de que a maioria dos escolares que trabalhavam analisados neste estudo eram mais velhos, entre 16 e 17 anos, sugerindo que eles poderiam estar em busca da autonomia financeira diante da crescente demanda de consumo.

Ao considerar a dependência administrativa da escola e a escolaridade materna como *proxy* de condição socioeconômica, observa-se que a população mais vulnerável é a maioria dos que trabalham. A escola pública possui um histórico de atendimento às demandas sociais da população, lidando com questões como a vulnerabilidade^20^, e, como o trabalho infantil está associado a contextos como esse, é compreensível que a maioria dos que trabalhavam eram matriculados nas escolas da rede pública, sendo elas mais demandadas por famílias de baixa renda^2^. Em relação à escolaridade materna, alguns estudos já apontaram uma associação entre o tempo de estudo da mãe e a ocorrência do trabalho infantil, demonstrando que níveis mais elevados de escolaridade materna atuam como fator de proteção ao trabalho infantil, devido ao entendimento da importância da educação para uma melhor qualidade de vida^21,22^ e melhores oportunidades no futuro, valorizando mais a educação em detrimento do trabalho^18^.

No que se refere às diferenças regionais do trabalho infantil, sabe-se que este sofre forte influência dos aspectos culturais, sendo que, em muitas dessas culturas, a oferta do trabalho infantil é considerada uma forma de educação^22^. A maior prevalência do trabalho infantil na região Sul do país pode estar ligada a esses fatores culturais, mas também às diferenças na densidade populacional entre as grandes regiões do país e também ao baixo dinamismo das atividades produtoras na região Norte e Nordeste em relação às regiões do Centro-Sul^19,22^. Cabe ainda destacar que os achados deste estudo quanto ao predomínio do trabalho infantil por região conflitam com outros disponíveis na literatura que evidenciam que o trabalho infantil ocorre mais nas regiões rurais, necessitando, assim, de aprofundamento nessa temática para compreender melhor essas diferenças^19,22^.

Acerca das chances mais elevadas para uso de drogas lícitas (cigarro e bebidas alcoólicas) e ilícitas (maconha, cocaína, *crack*, cola, loló, lança perfume, *ecstasy* ou outra) entre os adolescentes que trabalhavam evidenciadas neste estudo, acredita-se que esse cenário esteja associado ao efeito da adultização infantil sofrida por esses indivíduos, uma vez que passam a ter maior contato com pessoas mais velhas e, por quererem fazer parte do grupo, acabam adotando os mesmos comportamentos que elas, como, por exemplo, o consumo de drogas e vida sexual ativa^16^.

Quanto ao tabaco, sabe-se que o consumo dessa substância é um dos principais fatores de risco para o desenvolvimento das DCNT, ainda assim, no Brasil, em 2019, 22,6% (IC95% 21,7–23,4) dos adolescentes já haviam experimentado cigarro alguma vez na vida^23,24^. O fumo na adolescência está associado com piores condições econômicas, como o recebimento de um a três salários mínimos e o fato de estar cursando o ensino básico na rede pública do país^23,25^, além de aumentar a chance do uso de outras substâncias^23^.

No que diz respeito ao consumo de bebidas alcoólicas, é importante destacar que o uso de álcool entre os adolescentes também está relacionado a uma cultura de socialização, de atividade de lazer, busca por prazer, redução da timidez e aceitação dos amigos^26^ e que essa prática acarreta repercussões negativas imediatas e futuras para a saúde, como os efeitos sobre a memória, risco de acidentes e agravos externos, relações sexuais desprotegidas, influência sobre o humor e surgimento de DCNT na vida adulta^27^.

Já em relação ao uso de drogas ilícitas, a literatura aponta a ruptura de laços sociais, familiares, a depressão e as condições de vulnerabilidade, como as desigualdades de renda e pobreza, entre as causas resultantes no consumo dessas substâncias^28^, além do efeito do amadurecimento precoce pelo qual os adolescentes que trabalham passam^29,30^. As consequências desse uso podem acarretar prejuízos cognitivos, emocionais e sociais^31^.

Referente à maior chance de praticar atividade física por tempo suficiente relatada pelos adolescentes que trabalhavam, acredita-se que esse cenário pode ser explicado pelo tipo de trabalho exercido por esses indivíduos, que, por vezes, trabalham em locais como fazendas, indústrias e casas de família, exigindo maior movimentação e uso da força^32^. Ressalta-se que o indicador de atividade física usado neste estudo engloba todos os domínios de atividade física realizada, sendo o indicador preconizado pela Organização Mundial da Saúde para pesquisas entre escolares. A redução do comportamento sedentário, ou seja, ficar sentado por três ou mais horas por dia identificado nesse estudo pode estar associado à diminuição do tempo disponível para realizar atividades que contribuem para o sedentarismo como, por exemplo, o uso de telas como computadores, celulares e televisão^33^.

Os escolares que trabalham também apresentaram maior chance de consumo de alimentos não saudáveis, como guloseimas, salgados fritos e refrigerantes. Esse cenário se assemelha ao vivido pelos trabalhadores adultos, em que há a priorização de alimentos com maior praticidade na hora do preparo e de menor preço, sendo esses alimentos de baixo valor nutricional^34,35^. Alguns autores já destacam a importância da implementação de políticas públicas capazes de promover uma alimentação saudável, valorizando a cultura alimentar tradicional brasileira, além de propor medidas regulatórias que auxiliem a construção de um ambiente alimentar saudável^36^.

A principal limitação deste estudo diz respeito ao fato de que a PeNSE investiga apenas escolares regularmente matriculados e frequentes em escolas da rede pública e privada do Brasil, deixando de fora aqueles que não possuem esse vínculo educacional^13,37^, que, em geral, é ainda mais vulnerável e pode apresentar maior frequência do evento. Apesar disso, a PeNSE continua representativa para a população escolar do país, sendo um importante componente do sistema de vigilância em saúde dos adolescentes brasileiros^37^.

Em 2015, 16,9% dos escolares realizavam algum tipo de trabalho, sendo essa prática mais relatada pelos meninos, entre os mais velhos, pelos alunos de escolas públicas, pelos que possuíam menor escolaridade materna, e residentes da região Sul e Centro-Oeste do país. Esses indivíduos tiveram maiores chances de apresentar comportamentos considerados fatores de risco para as DCNTs como o uso de drogas e o consumo de alimentos não saudáveis, porém apresentando um cenário favorável em relação à prática de atividade física e redução do tempo sedentário.

Os achados deste estudo revelam a importância do acompanhamento dos comportamentos dos escolares brasileiros, uma vez que a exposição aos fatores de risco ainda na infância pode aumentam as chances de desenvolver algum tipo de DCNT na vida adulta. As evidências apresentadas apontam para os riscos imediatos e futuros que o trabalho na infância pode trazer para a saúde dos adolescentes e reitera a necessidade de ação imediata por parte do estado no combate a essa prática, bem como na redução das vulnerabilidades sociais e da pobreza.
